# PLGA Nanoparticles Grafted with Hyaluronic Acid to Improve Site-Specificity and Drug Dose Delivery in Osteoarthritis Nanotherapy

**DOI:** 10.3390/nano12132248

**Published:** 2022-06-30

**Authors:** Luana Zerrillo, Maria Rosa Gigliobianco, Domenico D’Atri, Joao Pedro Garcia, Fabio Baldazzi, Yanto Ridwan, Gastón Fuentes, Alan Chan, Laura B. Creemers, Roberta Censi, Piera Di Martino, Luis J. Cruz

**Affiliations:** 1 Translational Nanobiomaterials and Imaging (TNI) Group, Department of Radiology, Leiden University Medical Centrum, Albinusdreef 2, 2333 ZA Leiden, The Netherlands; zerrilloluana@gmail.com (L.Z.); fabio.baldazzi87@gmail.com (F.B.); gastonfe@gmail.com (G.F.); 2 Percuros B.V., Zernikedreef 8, 2333CL Leiden, The Netherlands; roselili87@gmail.com (M.R.G.); achan@percuros.com (A.C.); 3 School of Pharmacy, University of Camerino, Via Madonna delle Carceri 9, 62032 Macerata, Italy; roberta.censi@unicam.it; 4 Department of Biotechnology and Food Engineering, Technion Israel Institute of Technology, Haifa 3200, Israel; domenicodatri88@gmail.com; 5 Department of Orthopedics, Utrecht Medical Center, Heidelberglaan 100, 3584CX Utrecht, The Netherlands; joaopedromgarcia@gmail.com (J.P.G.); l.b.creemers@umcutrecht.nl (L.B.C.); 6 Department of Radiology & Nuclear Medicine and Department of Molecular Genetics, Erasmus University Medical Center, Dr. Molewaterplein 40, 3015 GD Rotterdam, The Netherlands; r.ridwan@erasmusmc.nl; 7 Department of Ceramic and Metallic Biomaterials, Biomaterials Center, University of Havana, Ave. Universidad e/G y Ronda, Vedado, Plaza, La Habana 10400, Cuba; 8 Department of Pharmacy, Università “G. d’Annunzio” di Chieti e Pescara, Via dei Vestini 1, 66100 Chieti, Italy; piera.dimartino@unich.it

**Keywords:** drug delivery, gold NPs, intra-articular injection, molecular imaging, nanotechnology

## Abstract

Nanoparticles (NPs) have a tremendous potential in medicinal applications, and recent studies have pushed the boundaries in nanotherapy, including in osteoarthritis treatments. The aim of this study was to develop new poly(lactide-co-glycolide) (PLGA) nanoparticles (NPs) surfaces decorated with hyaluronic acid (HA) to enhance targeted drug specificity to the osteoarthritic knee joint. HA was selected since it binds to specific receptors expressed in many cells, such as the cluster determinant 44 (CD44), a major receptor of chondrocytes, and because of its function in the synovial fluid (SF), such as maintenance of high fluid viscosity. The PLGA polymer was grafted to sodium hyaluronate using dimethoxy-PEG (PLGA-HA) and compared with control PLGA NPs (not grafted). NPs were characterized by 1H-NMR and IR spectroscopy. Then, near-infrared (NIR) dye and gold (20 nm) were encapsulated in the formulated NPs and used to access NPs’ performance in in vitro, in vivo, and ex vivo experiments. To test the NPs’ CD44 receptor specificity, an antibody assay was performed. All NPs presented a size in the range viable for cell-uptake, no cytotoxicity to chondrocytes was registered. Although all the NPs had a high capacity to be absorbed by the cells, PLGA-HA NPs showed significantly higher affinity towards the chondrocytic C28/I2 cell line. In conclusion, PLGA NPs grafted to sodium hyaluronate showed increased binding to cartilage cells and tissue and enhanced accumulation at the target site. Thus, this study presents a safe drug-delivery system with improved receptor specificity, which may represent an advantageous alternative to current nanotherapies.

## 1. Introduction

Osteoarthritis (OA) is a progressive degenerative disorder of the articular cartilage. It is characterized by the rupture and subsequent loss of joint cartilage that causes synovial inflammation and hyperplasia of the joint capsule, with the consequent impaired mobility and pain. It is estimated that about 60% of men and 70% of women aged 60 years and older have OA, making it the most common chronic joint disease [1]. OA is related to multiple factors, such as congenital anomalies, hormonal alterations, inflammatory diseases, repeated trauma or surgery of joint structures, and obesity [2,3]. The most common and current treatments for OA are based on palliative care and consist of analgesics (paracetamol) and anti-inflammatory drugs (NSAIDs) and intra-articular injections (IA) of hyaluronic acid (HA) or corticosteroids [4]. However, the efficacy of these treatments is relatively low due to the rapid clearance and consequently short retention time in the synovial joint [5], which increases the risk of inflammation. Therefore, it is imperative to overcome the limitations of current treatments because their improvement would increase the retention time of synovial fluid and/or the target specificity.

This study concerns grafting hyaluronic acid (HA) onto PLGA nanoparticles to improve the tissue specificity of drug delivery. Due to its endogenicity in synovial fluid, HA is known to reduce frictional force within the knee joint by acting as a viscoelastic lubricant. It has the ability to bind specifically to CD44, a type I transmembrane receptor protein. It is abundant in human articular chondrocytes and promotes proliferation by binding to HA [6,7,8]. PLGA, on the other hand, is a biocompatible and biodegradable polymer used as a support matrix in the development of active drug delivery systems with selective targeting and release. Nanoparticles (NPs) with a hydrophobic core of PLGA coated on the surface with hydrophilic HA could be formed using the amphiphilic properties of the grafted polymer. This system may aid in improving biodistribution in the knee joint, allowing drugs to be released intracellularly and enhancing chondrocyte growth [5,9,10]. In ex vivo and in vivo studies, gold was chemically anchored in PLGA NPs as a contrast agent to detect its presence by computed tomography (CT) [11,12]. Furthermore, due to their anti-inflammatory and anti-angiogenic properties, gold nanoparticles have been used as a therapeutic treatment for rheumatic diseases [13,14].

The primary goal of this study was to create a proof of concept for the use of HA-coated PLGA NPs in the treatment of OA. NPs of PLGA coated with HA were created and physically and chemically characterized for this purpose. Using human cartilage, performance was assessed in vitro, in vivo, and ex vivo using molecular imaging. In contrast, PLGA NPs that had not been grafted with HA were used.

## 2. Materials and Methods 

### 2.1. Materials

Poly(D, L-lactide-co-glycolide) (PLGA RG 503H, lactide:glycolide 50:50, MW 39000), N,N′-dicyclohexylcarbodiimide (DCC), 1-ethyl-3-[3-(dimethyl amino) propyl] carbodiimide (EDC), thionyl chloride 99.9%, poly(ethylene glycol) dimethyl ether (dmPEG) (MW: 2000), CTAB (cetyltrimethylammonium bromide), and HAuCl_4_•3H_2_O (99%) were purchased from Sigma-Aldrich (Merck KGaA, Darmstadt, Germany). Sodium hyaluronate HA20K-5, Research Grade (MW: 21–40 KDa), was supplied by Lifecore Biomedical LLC (Chaska, MN, USA).

### 2.2. Polymer Synthesis

#### 2.2.1. Synthesis and Characterization of PLGA-Grafted Sodium Hyaluronate Copolymer 

The PLGA-HA graft copolymer was obtained in three reaction steps. In the first step, 1 g of sodium hyaluronate (MW: 21–40 kDa) and 5 g of dmPEG were mixed in 25 mL of DMSO to prepare the graft copolymer according to the method previously described by Lee et al. [15]. To activate PLGAm, 0.018 mmol of PLGA was first dissolved in methylene chloride. Then, 7.0 mg of DCC and 4.0 mg of NHS were added at room temperature in an inert atmosphere (dry N_2_) for 24 h (PLGA/NHS/DCC stoichiometric molar ratio: 1/2/2). The resulting solution was filtered and precipitated into ice-cold diethyl ether and dried thoroughly in vacuo [16]. Finally, 1 g of sodium hyaluronate/dmPEG complex was dissolved in 10 mL of anhydrous DMSO at 80 °C for 2 h under dry N_2_ and 60.0 mg of activated PLGA in 2.5 and 3.0 mL of anhydrous DMSO, respectively. PLGA was added dropwise to the HA solution under dry N_2_. The polymer mixture was stirred for 2 days at room temperature. It was then dialyzed in DMSO for 1 day and in deionized water for 3 days using dialysis membrane (MWCO: 12–14 kDa) to remove by-products. PLGA-HA copolymers were obtained in quantitative yield and analyzed by ^1^H-NMR, IR spectroscopy, and the cetyltrimethylammonium bromide (CTAB) turbidimetric assay.

#### 2.2.2. Chemical Characterization of the Synthesized Polymer

^1^H-NMR (400 MHz for ^1^H) spectra were recorded on a Varian Mercury Plus NMR 400 MHz Oxford as 400 Magnet spectrometers (∂ = 2.54 ppm for DMSO) (Varian Medical Systems, Palo Alto, CA, USA). The infrared (IR) spectrum was recorded from 4000 to 400 cm^−1^ with a PerkinElmer Spectrum 100 FT-IR instrument (Perkin Elmer, Rotterdam, The Netherlands). This analytical technique was used to identify the characteristic of the synthesized PLGA-HA copolymer. 

#### 2.2.3. Quantification of Hyaluronic Acid 

The amount of HA grafted to the PLGA-HA copolymer was determined by the cetyltrimethylammonium bromide (CTAB) turbidimetric assay: 2.5 g of CTAB was dissolved in 100 mL of 2% (*w/v*) NaOH, and the CTM reagent was obtained using a previously published method [17,18], with some modifications. Briefly, 50 µL of HA standard solution and PLGA-HA polymer samples were introduced into 96-well plates with 50 µL of 0.1 M phosphate buffer at pH 7 and incubated at 37 °C for 15 min. Then, 100 µL of CTM reagent was added to each well and the plates were incubated again for 10 min, at 37 °C. The absorbance of the solution was read at 600 nm with a microplate reader (FLUO star Omega, BMG LABTECH, Pero, Italy). The amount of unconjugated hyaluronic acid was calculated from the linear regression of the standard curve obtained from HA solutions of known concentrations.

### 2.3. Preparation of Gold Nanoparticles

Gold NPs (HAuCl_4_) were obtained through the reduction of tetra chloroauric acid by aqueous sodium citrate. A solution of 2.2 mM sodium citrate in Milli-Q water was heated to 170 °C under vigorous stirring. Then, 25 mM of HAuCl_4_ was added dropwise in the sodium citrate solution. The solution switched from yellow to brilliant red, due to the formation of monodisperse spherical particles. The gold particles were collected by precipitation using a centrifuge at 3000 rpm and at room temperature for 30 min.

### 2.4. Preparation of PLGA and PLGA-HA NPs

Two types of NPs were generated: (1) control PLGA NPs and (2) HA-grafted PLGA NPs. All NPs were obtained with the double-emulsion solvent, water-oil-water (W/O/W) evaporation method and were doped with NIR dye and gold NPs. The oil phase containing the polymer (PLGA or PLGA-HA) and the NIR dye was added dropwise to the first aqueous phase containing gold in Milli-Q water. The emulsion was mechanically stirred for 5 min with an Ultra-Turrax IKA T-25 (IKA^®^-Werke GmbH & Co., Staufen, Germany). The obtained water-to-oil emulsion was then added dropwise to the second aqueous phase containing 0.5% *w/v* PVA as an emulsifying agent. The water-oil-water emulsion was continuously stirred at 24,000 rpm for 20 min with the Ultra-Turrax until the organic solvent partitioned into the aqueous phase and then evaporated. The suspended NPs that were formed were stirred overnight under magnetic stirring (IKA^®^ RCT basic IKAMAG^TM^, IKA^®^-Werke GmbH & Co., Staufen, Germany) at 4 °C and at moderate speed to ensure complete evaporation of the solvent. The polydisperse NPs’ suspension was separated by ultracentrifugation (14,000 rpm/4 °C/30 min). Then, they were washed four times with Milli-Q water to remove excess PVA, resuspended in Milli-Q water, and lyophilized. For the preparation of FITC dye-loaded PLGA NPs and PLGA-HA NPs, a protocol similar to that described above for the NIR dye was used.

### 2.5. Characterization of Nanoparticles

#### 2.5.1. Encapsulation Efficiency Analysis of NIR Dye 

To determine the encapsulation efficiency (EE) of the NIR dye in the NPs, they were dissolved in 0.8 M NaOH. In addition, 5 mg each of PLGA NP and PLGA-HA NP were dissolved separately in 0.5 mL of NaOH 0.8 M overnight at 37 °C. Subsequently, all NP solutions were centrifuged at 12,000 rpm and at room temperature for 20 min, and the supernatants were collected. Dye content was measured using the Odyssey Infrared Imager 9120 (LI-COR) scanner (The LabWorld Group, Hudson, MA, USA) at a wavelength of 800 nm. The encapsulation efficiency of the NIR dye was calculated according to Equation (1):

(1) $$EE\%=\frac{Amount\;of\;colorant\;in\;the\;formulation}{Amount\;of\;colorant\;used\;for\;the\;formulation}\times 100$$

where the *Amount of colorant in the formulation* is the amount of NIR dye loaded on the NPs. The *Amount of colorant used for the formulation* is the amount of NIR dye added in the NP preparation.

#### 2.5.2. Encapsulation Efficiency Analysis of FITC Dye

Five mg of dry NPs was dissolved in 0.8 M NaOH to study the encapsulation efficiency of the fluorescein isothiocyanate (FITC) dye, as described above. Control solutions containing PLGA NPs and PLGA-HA NPs were centrifuged at 12,000 rpm for 20 min at room temperature, and all the supernatants were collected. FITC dye content was then measured with an Amersham Biosciences Ultrospec 2100 pro UV/Vis spectrophotometer (American Laboratory Trading, East Lyme, CT, USA). The amount of FITC dye was calculated from the linear regression of the standard curve obtained from the FITC solution.

#### 2.5.3. Encapsulation Efficiency Analysis of Gold 

Similarly, as described above, 5 mg of NPs was dissolved in 0.8 M NaOH. NPs were centrifuged at 12,000 rpm for 20 min at room temperature and supernatants were collected. Absorbance was read at 500 nm with the VERSAmax Tunable Microplate Reader (SoftMAX Pro v5.4.1, Molecular Devices, San José, CA. USA). The amount of gold was calculated from the linear regression of the standard curve obtained from the gold standard solution.

#### 2.5.4. Particle Size and Charge Surface 

Dynamic light scattering (DLS) was used to determine the average size and polydispersity index (PDI) of NPs dispersed in Milli-Q water at 25 °C and an angle of 90° (Zetasizer Nano S90, Malvern Instruments, Worcestershire, United Kingdom). The stability or aggregation of the NPs was determined by the zeta-potential using the same equipment. All measurements were performed in triplicate.

#### 2.5.5. Particle Surface and Morphology 

Transmission electron microscopy (TEM) was used to visualize and characterize the morphology and contrast the NPs’ size. A formvar film attached to a copper grid (100 mesh) was coated with carbon and hydrophilized by glow discharge (30 s/25 mA). Three μL of the particle’s solution was added to the grid for 1 min, removing excess suspension by transfer. The grid was then stained for 1 min with uranyl acetate (2.3% in distilled water), removing the excess in a similar manner as described above. Subsequently, the grid was air-dried, and images were taken on a FEI T12 Spirit BioTwin (FEI Company; Hillsboro, OR, USA) equipped with an OneView Camera Model 1095 (Gatan; Pleasanton, CA, USA) operated at 120 keV. The sample was imaged at 3 μm under focus with binning 2 on a 4k × 4k Eagle CCD camera (Los Altos, CA, USA) at 18,500×.

### 2.6. Release Studies of NIR Dye and Gold from NPs

#### 2.6.1. Release Study of NIR Dye and Gold in PBS 

The gold and NIR dye release studies were performed for one month, as previously described [19]. Briefly, 5 mg of lyophilized PLGA and PLGA-HA NPs were dissolved in 5 mL of phosphate buffered saline (PBS). The solutions containing NPs were gently shaken at 25/27 °C. At the 0, 3-, 9-, 12-, 14-, 17-, 23-, 27-, and 30-day time points, NPs’ solutions were centrifuged for 20 min at 12,000 rpm and 150 μL of supernatant was collected and replaced with 150 μL of fresh PBS solution. Each aliquot was then quantified with the Odyssey™ scanner (The LabWorld Group, Hudson, MA, USA) using the 800 nm channel for NIR dye visualization. For gold quantification, collected supernatants were read at 500 nm with the VERSAmax Tunable Microplate Reader (SoftMAX Pro v5.4.1, (Molecular Devices, San José, CA, USA)). 

#### 2.6.2. Release Studies of NIR Dye and Gold in Synovial Fluid 

A NIR and gold dye release study was performed in human synovial fluid (SF) for all NPs (PLGA and PLGA-HA). Synovial fluid was obtained from anonymous patients who underwent knee replacement surgery at the Department of Orthopedic Surgery at Leiden University Medical Center. Here, 1 mg of lyophilized NPs was dissolved in 1 mL of SF. As described above, the NPs’ solutions were kept under gentle agitation at 25/27 °C. At the 0, 3-, 9-, 12-, 14-, 17-, 23-, 27-, and 30-day time points, NP-containing solutions were centrifuged for 20 min at 12,000 rpm and 100 μL of supernatant was collected and replaced with 100 µL of fresh SF. The collected supernatants were quantified with the Odyssey™ scanner using the 800 nm channel for NIR dye visualization. For gold quantification, the collected supernatants were measured at 500 nm with the Molecular Devices VERSAmax Tunable Microplate Reader (SoftMAX Pro v5.4.1).

### 2.7. In Vitro Characterization of Nanoparticles

#### 2.7.1. Cell Culture

The C28/I2 human chondrocyte cell line is used as a model to study normal and pathological cartilage repair [20,21]. C28/I2 cells were grown in 75 cm flasks in 1:1 Dulbecco’s modified Eagle’s medium (DMEM)/F12 medium (Gibco, ThermoFisher Scientific, Waltham, MA, USA) with 10% (*v/v*) fetal bovine serum (FBS; Life Technology, ThermoFisher Scientific, Waltham, MA, USA) at 37 °C and an atmosphere of 95% air and 5% CO_2_. The medium was replaced every 48 h. Cells were sub-cultured for experiments after growing to approximately 80–90% confluency. 

#### 2.7.2. Cell Metabolic Assay (MTS)

Cell viability of all NPs was assessed by the MTS assay with the C28/I2 cell line using 5 × 10^4^ cells/well in 96-well plates. Different concentrations (10, 20, 40, and 80 µg/mL) of NPs were added to each well after 24 h of incubation at 37 °C to study the metabolic activity at 24, 48, and 72 h of incubation. As a positive cytotoxicity control, cells were treated with 50% of DMSO. Untreated cells were used as a negative control. Subsequently, 20 µL of 3-(4,5-dimethylthiazol-2-yl)-5-(3-carboxymethoxyphenyl)-2-(4-sulfophenyl)-2H-tetrazolium (MTS) (Promega, ThermoFisher Scientific, Waltham, MA, USA) was added to each well and incubated for 1 h at 37 °C. Absorbance (λ_ex_ = 590 nm) was measured (VERSAmax Tunable Microplate Reader, Molecular Devices, San José, CA, USA). The assay was evaluated according to the manufacturer’s instructions. The metabolic activity of the cells in each condition is expressed as a percentage increase relative to untreated controls.

#### 2.7.3. Cell Binding Analysis by Odyssey 

Here, 2.5 × 10^4^ C28/I2 cells/well were seeded in a 96-well microplate (Greiner Bio-One, Kremsmünster, Austria) and maintained at 4 °C with 40 µg/mL of PLGA and HA-PLGA NPs for 0, 1, 2, 4, 8, and 24 h. Subsequently, cells were washed twice with PBS, fixed with 2% paraformaldehyde in PBS for 15 min, and rinsed again with PBS. They were then stained with TO-PRO^®^-3 iodide dye (Molecular Probes, ThermoFisher, Marietta, OH, USA), which stains the cell nucleus detectable at 700 nm. The plate was analyzed by the Odyssey Infrared Imager 9120 (LI-COR) scanner (The LabWorld Group, Hudson, MA, USA) using an 800 and 700 nm channel used for visualization of NIR-loaded NPs and cells, respectively.

### 2.8. NPs Uptake Studies 

#### 2.8.1. Uptake Characterized by Odyssey

C28/I2 cells (2.5 × 10^4^/well) were seeded in a 96-well cell culture microplate (Greiner Bio-One, Kremsmünster, Austria) and incubated with 40 µg/mL of PLGA and PLGA-HA NPs for 1, 2, 4, and 8 h. Then, they were washed twice with PBS, fixed for 15 min with paraformaldehyde (2% in PBS), rinsed with PBS, and stained with TO-PRO^®^-3 iodide stain (Molecular Probes, ThermoFisher, Marietta, OH, USA), which makes the cell nucleus detectable at 700 nm. The plate was analyzed by the Odyssey Infrared Imager 9120 scanner (The LabWorld Group, Hudson, MA, USA) using an 800 and 700 nm channel used for visualization of NIR-loaded NPs and cells, respectively.

#### 2.8.2. Uptake Characterized by Flow Cytometry

C28/I2 cells (7 × 10^5^/well) were seeded in a 24-well plate (ThermoFischer, Waltham, MA, USA) and grown overnight. To demonstrate that PLGA-HA NPs were absorbed through the CD44 receptor, the next day, the medium was replaced with fresh medium supplemented with hyaluronic acid (20 mg/mL) or with anti-CD44 antibody (ThermoFischer catalogue, dilution 1:500) to block the CD44 receptor. After 1 h of incubation, cells were washed three times with BSA/PBS (0.5%). Then, they were incubated with 5, 10, 20, and 40 μg/mL of FITC-PLGA and FITC-PLGA-HA NPs for 1 h, washed three times with BSA/PBS (0.5%), collected, and measured. The FITC-positive signal was measured using non-specific cells as a background. Analysis was performed with a BD LSR-II flow cytometer (BD Biosciences, Franklin Lakes, NJ, USA) and results were analyzed with FlowJo 10 (FlowJo LLC, Ashland, OR, USA) using mean fluorescence intensity as the study parameter.

#### 2.8.3. Uptake Characterized by Confocal Microscopy

To assess intracellular uptake of NPs, 26,000 C28-I2 cells/cm^2^ were seeded on Cellview™ plates (Greiner Bio-one, Kremsmünster, Austria), using DMEM/F-12 supplemented with 10% fetal bovine serum (FBS, Biowest, Nuaillé, FR), 0.2 mM ascorbic-2-phosphate (Sigma-Aldrich, The Netherlands), and 100 µg/mL penicillin and streptomycin (Gibco, ThermoFischer, Waltham, MA, USA). Cells were cultured for 24 h in a humidified incubator at 37 °C and 5% CO_2_. Subsequently, 40 µg/mL of all NPs were added to the cells and incubated for an additional 24 h in un-supplemented DMEM/F-12. Cells were then fixed with formalin for 20 min, followed by 0.2% PBS-Triton for 20 min, and blocked with 5% BSA/PBS for 30 min. Subsequently, they were stained with 2.5 µg/mL of phalloidin-TRITC (Millipore-Sigma, Burlington, MA, USA) and 100 ng/mL of DAPI (Millipore-Sigma, Burlington, MA, USA) for 1 h. Between each step, cells were washed three times with 0.05% Tween/PBS. Images were acquired using a Leica SP8X confocal microscope (Leica Microsystems, Wetzlar, Germany) with a 63×/1.4 oil immersion objective. Image processing and analysis were performed with Fiji software, version 1.50 (National Institutes of Health, Bethesda, MA, USA).

### 2.9. In Vivo Characterization of NPs

#### 2.9.1. Animals and Experimental Design

Animal procedures were performed at Leiden University Medical Center and approved by the Animal Welfare Committee under agreement number 12036 under the European Community Council Directive, 1986. A total of 9 12-week-old male C57BL/6Jico mice were purchased from Charles River Labs (Wilmington, MA, USA). They were divided into three equal groups: three mice were kept as a control group and did not receive NPs (group 1), and the remaining six mice were divided into two groups and intra-articular injections were administered, 2.0 mg/mL of PLGA NPs (group 2) and PLGA-HA NPs (group 3). All mice (groups 1, 2, and 3) were sacrificed 15 days after NP injections and their hind paws were analyzed by μCT scans.

#### 2.9.2. NPs Retention Time in the Mouse Knee Joint

The IVIS Spectrum in vivo imaging system (Perkin Elmer, Waltham, MA, USA) was used to measure the retention time of fluorescent NPs in the knee joint. Mice were scanned after 1, 7, and 15 days of intra-articular NP injection after they were anesthetized with isoflurane. Image data were analyzed using Living Image 4.3.1 software (Perkin Elmer, Waltham, MA, USA). Fluorescence images were acquired using a 710 nm excitation filter and 760, 780, 800, 820, and 840 nm emission filters. Both hindlimbs were scanned to monitor background tissue fluorescence.

#### 2.9.3. Detection of NPs in Mouse Knee Joint by CT Scans 

Ex vivo μCT images were acquired using a 10 mm field of view (90 kV/160 mA, 4.5 min) at 20 μm resolution. Scans were compiled into 3D images using Analyze Direct™ software (AnalyzeDirect, Inc., Overland Park, KS, USA). A global threshold was applied by visual inspection to all µCT scans.

### 2.10. Ex Vivo Characterization of NPs

#### NPs Targeting Studies on Human Cartilage Explants 

The studied tissue explants were prepared from the articular cartilage of patients undergoing total knee arthroplasty. The anonymous use of redundant tissue for research purposes is part of the standard treatment agreement with patients at Utrecht University Medical Center and was carried out under UMCU Biobank Review Board Protocol No. 15-092. Explants (Ø = 4 mm) were harvested using a biopsy punch and subsequently washed in PBS containing 100 U/mL of penicillin and 100 mg/mL of streptomycin (15140122, Gibco, ThermoFisher Scientific, Waltham, MA, USA). Untreated explants were used as controls, while 10 ng/mL of tumor necrosis factor-α (TNF-α) was added to positive controls for 15 days to mimic inflammation. Subsequently, the cartilage explants were placed in a 96-well plate and incubated with 40 μg/mL of PLGA or PLGA-HA NPs in 250 μL of DMEM/F12 medium (Gibco Cell Culture Medium, ThermoFisher Scientific, Waltham, MA, USA) with 10% (*v*/*v*) fetal bovine serum (FBS; Life Technology, Waltham, MA, USA) culture medium for 24 and 48 h. After washing healthy explants and positive controls five times with PBS, cartilage explants were scanned with µCT using a 5 mm field of view (90 kV/160 mA, 4.5 min) with 10 μm resolution. Scans were compiled into 3D images using Analyze Direct™ software (Overland Park, KS, USA). The global threshold was applied to all μCT scans and was determined by visual inspection. Then, human cartilage explants were embedded in O.C.T. compound, cut with a microtome, and imaged by fluorescence microscopy (Leica DMRA fluorescence microscopy, Leica Microsystems, Wetzlar, Germany).

### 2.11. Statistical Analysis

All experiments were performed in triplicate, except for in vivo studies. For statistical analysis, GraphPad Prism version 5 software (GraphPad, San Diego, CA, USA) was used. Data were analyzed by *t*-test and two-way analysis of variance (ANOVA). In all analyses, a significant difference was inferred at α < 0.05.

## 3. Results and Discussions 

### 3.1. Synthesis and Characterization of PLGA-Grafted Hyaluronate Copolymers

**Figure 1:**
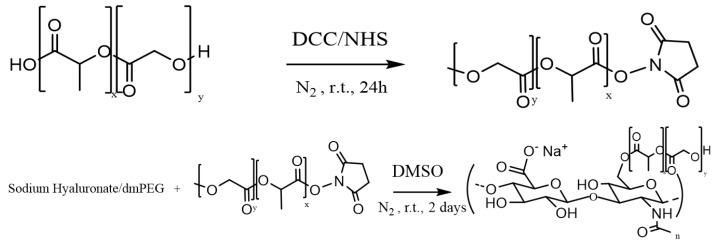
Schematic synthesis for PLGA-HA polymer.

One of the main objectives of this study was to obtain NPs with a PLGA core grafted with HA. The PLGA-HA copolymer was synthesized in 57% yield based on the synthetic scheme shown in Figure 1. 

**Figure 2:**
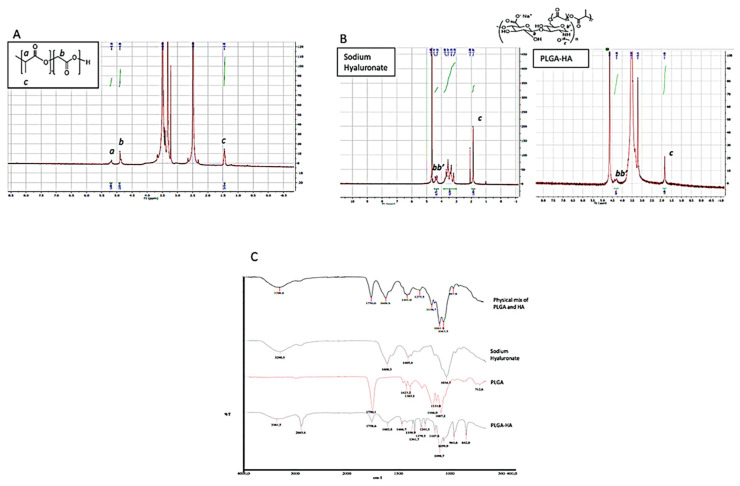
Characterization of the synthesized PLGA-HA polymer. (**A**) 1H-NMR spectrum in DMSO of PLGA-HA (**B**) From left to right side, respectively, ^1^H-NMR spectra in D_2_O of sodium hyaluronate and PLGA-HA (**C**) From top to bottom, IR spectrums of the physical mix of PLGA and HA, HA, PLGA, and PLGA-HA.

The chemical characterization of the synthesized copolymer was performed by ^1^H-NMR in DMSO and D_2_O (Figure 2A,B) and by IR spectroscopy (Figure 2C). HA, which is not soluble in organic solvents, was physically complexed with dmPEG to solubilize it in anhydrous DMSO. The terminal carboxyl group of PLGA was activated by the DCC coupling reaction. Activated PLGA was then conjugated to the HA/dmPEG complex through the hydroxyl groups of HA.

The NMR spectrum highlights the peaks of PLGA at 5.20 ppm (m, CH of PLGA, Figure 2(Aa)), 4.90 ppm (m, CH_2_ of PLGA, Figure 2(Ab)), and 1.45 ppm (s, CH_3_ of PLGA, Figure 2(Ac)). The presence of conjugated HA via an ester linkage was confirmed by the peak of HA acetyl (-NHCOCH_3_) at 1.85 ppm (Figure 2(Bc)) and by IR spectroscopy (Figure 2C). HA exhibited broad OH stretching vibration at 3200–3600 cm^−1^, while for PLGA the stretching of CH (2950 cm^−1^) and C=O (1749 cm^−1^) was evident (Figure 2C). The synthesized PLGA-HA exhibited mixed spectra of HA and PLGA. The NMR signals are mixed, which suggests that there was coupling of PLHA-HA, although slightly displaced and decreased in intensity due to the association between both molecules with special emphasis in 4.30 ppm (d, CH, C_1_ of copolymer HA saccharides in the, Figure 2(Bbb’)). The spectra of PLGA-HA showed -OH stretch of HA at 3360 and 3365 cm^−1^, respectively. The presence of PLGA in the synthesized polymer was confirmed by -CH stretch of PLGA-HA at 2884.8 and 2885.8 cm^−1^ and C=O at 1759 and 1758 cm^−1^. The spectrum of synthesized polymer was also compared with a simple physical mix of PLGA and HA; in the former, the bending at 1608 cm ^−1^ was clearly evident (Figure 2C), confirming the chemical conjugation of HA to the PLGA polymer. With the CTAB assay, which is a cation surfactant normally used to quantify polysaccharides [18], it was possible to demonstrate that the amount of HA coupled to the PLGA was 80%. 

Compared to the current literature, the presented methodology is robust and economically feasible for the synthesis of PLGA copolymers grafted with sodium hyaluronate. The main advantages of the presented methodology are: (i) possible coupling of two polymers, (ii) possibility of industrial scaling due to the soft conditions of the reaction and the short activation time, and (iii) use of a non-toxic coupling agent specific for the carboxyl group of PLGA-g-HA, a specific CD44 receptor, available at a low cost and stable for long-term storage.

### 3.2. NPs Formulation and Characterizations

PLGA and PLGA-HA copolymers were used to prepare NPs using the solvent evaporation double-emulsion method. This method allowed the formation of hydrophobic PLGA chains in the core of the NPs and a hydrophilic crown of HA on the surface of the NPs [10]. One of the most important challenges in the field of drug delivery is the development of potential targeting systems that can selectively interact with receptors expressed on specific cells [22]. This study demonstrates that HA can be used as a ligand on the surface of NPs to target a specific site, such as human cartilage. The mean size of the PLGA NPs was 228 ± 1 nm and of the PLGA-HA NPs was 200 ± 2 nm, with a polydispersity index of 0.31 ± 0.05 and 0.6 ± 0.2 (Table 1). The similar average indicated that the presence of HA did not affect the size of the NPs. It is important to note that the size of all the prepared NPs is in the appropriate range for their activity by cellular uptake [23].

**Figure 3:**
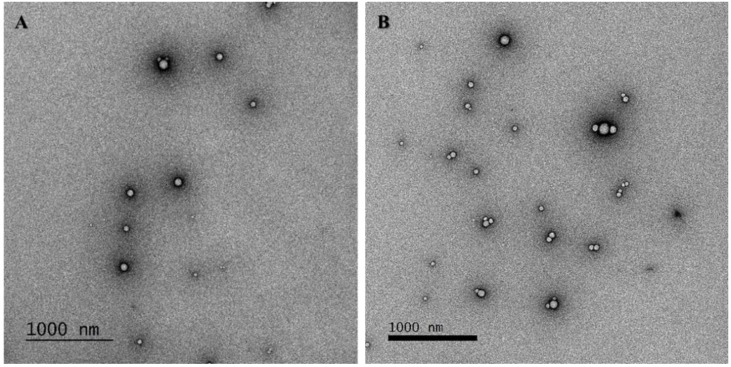
NPs’ morphology characterization. Representative TEM images of (**A**) PLGA NPs and (**B**) PLGA-HA NPs.

The ζ-potential data indicates that both the PLGA and PLGA-HA NPs were negatively charged. This was mainly due to the presence of carboxyl groups on both surfaces, and concentration increased in the case of HA (Table 1). The small increase in negative charge in the PLGA-HA NPs relative to the uncoated ones probably depends on the presence of a higher density of carboxyl groups by the grafting of HA on the surface of the NPs. The higher zeta-potential creates a repulsive force that helps stabilize the colloidal suspension and prevents NPs’ aggregation [24]. TEM analysis revealed that PLGA NPs and PLGA-HA NPs exhibit a smooth surface and uniform size distribution (Figure 3A,B). This is in agreement with the results obtained by DLS [25]. 

PLGA NPs and PLGA-HA NPs had high NIR dye encapsulation efficiencies, 84.3% and 65.3%, respectively (Table 1). The trend was maintained in the case of gold encapsulation, at 16.1% (PLGA NPs) and 8.4% (PLGA-HA NPs). Gold detection was recorded by μCT on film (see Supplementary Material, Video S1). For all charged compounds (NIR dye, gold, and FITC dye), a lower encapsulation efficiency was observed for PLGA-HA NPs, which may be due to the presence of hyaluronic acid on the surface of the NPs. 

#### Release Studies 

**Figure 4:**
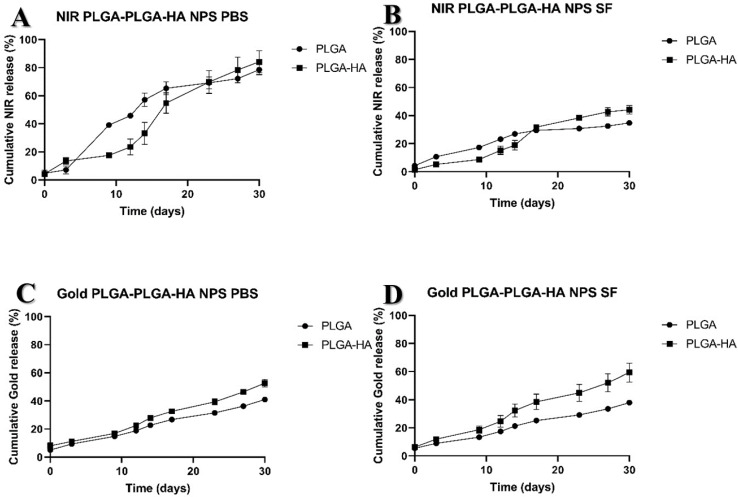
Release study of NIR dye and gold particles from PLGA NPs and PLGA-HA NPs in PBS and in SF. (**A**) Cumulative release of NIR dye in PBS, (**B**) cumulative release of NIR dye in SF, (**C**) cumulative release of gold in PBS, and (**D**) cumulative release of gold in SF.

The NIR dye release study of PLGA and PLGA-HA NPs in PBS showed similar patterns, with about 18% of the NIR dye released during the first day. However, after 10 days, PLGA-NPs released nearly 40% of the NIR dye, while PLGA-HAs approximately 20%, indicating a slower release for the latter compared to PLGA-NPs (Figure 4A). This reflects a biphasic release process induced mainly by the surface of the NPs. NIR could diffuse easily in a first phase, followed by a slower release in a second phase (up to 20 days), which could be caused by the interaction of graft charges that cause repulsion in collusion with the increase in the NIR dye concentration in the solution under study that would tend to a progressively increasing equilibrium in the system. 

All NPs showed a similar slow-release profile of the NIR dye in SF (Figure 4B). At the end of 30 days, PLGA NPs had released on average approximately 40% of the NIR dye, while PLGA-HA NPs released ca. 50%. Gold NPs in both media, PBS and SF, also showed a similar profile over time for both PLGA and PLGA-HA (Figure 4C,D). However, a large difference for the 30-day NIR release of 70% and 80% versus 30% and 40% for HA conjugated and unconjugated NPs, respectively, was observed between SF and PBS media. This is most likely due to the hydrophobicity of the NIR label in combination with the SF containing endogenous negatively charged HA. 

### 3.3. Characterization of the NPs in the In Vitro Experiments

#### 3.3.1. Cellular Metabolic Assay and Chondrocytes Binding Assay

**Figure 5:**
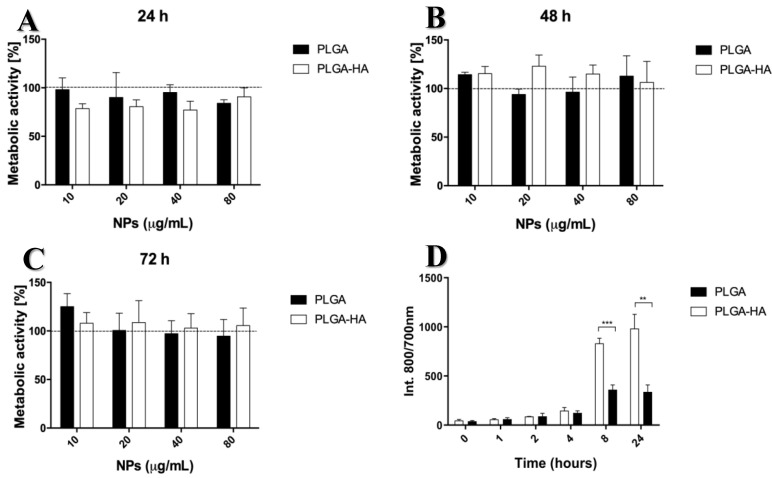
Cell metabolic activity assay (MTS) of the 2 different NPs incubated with the C28/I2 cell line at different concentrations. Metabolic cell activity was measured at three time points: (**A**) 24 h, (**B**) 48 h, and (**C**) 72 h. For each assay, the positive control was obtained using DMSO 50% and the negative control was obtained with only the cells. The negative controls are indicated by the black horizontal line. (**D**) Binding assay of PLGA and PLGA-HA NPs incubated with the C28/I2 cell line at the concentration of 40 µg/mL, measured at different time points after NPs’ incubation. There was no statistically significant difference (*p* > 0.05) between PLGA NPs and PLGA-HA NPs at time points 0, 1, 2, and 4 h. ** Indicates statistically significant difference (*p* < 0.05) between PLGA NPs and PLGA-HA NPs after 8 h of NPs’ and cells’ incubation. *** Indicates statistically significant difference (*p* < 0.001) between PLGA NPs and PLGA-HA NPs after 24 h (*n* = 3).

The results of the MTS assay revealed that neither NP type was cytotoxic to cells at the time points studied. In the first 24 h, all concentrations of PLGA-HA NPs exhibited a statistically non-significant reduction in metabolic activity, followed by an increase over time, probably due to cell proliferation (Figure 5A). 

In recent years, many studies have shown that PLGA is highly biocompatible and suitable for in vitro and in vivo treatment, justifying its increasing presence in various FDA-approved drug formulations. In aqueous medium, this copolymer degrades into lactic acid and glycolic acid, which enter the tricarboxylic acid cycle of chondrocytes and are eliminated as carbon dioxide and water [26]. This may explain the increase in cellular metabolic activity after 48 and 72 h of incubation with NPs and C28/I2 (Figure 5B,C). 

To study the binding capacity of PLGA and PLGA-HA NPs to chondrocytes, NPs were incubated with C28/I2 cells, and their fluorescence intensity was measured at different times. There was a clear increase in binding capacity over time. Initially (0, 1, 2, and 4 h of incubation), there were no significant differences between PLGA NPs and PLGA-HA NPs. However, after 8 and 24 h, PLGA-HA NPs showed a significantly higher binding capacity compared to PLGA NPs (Figure 5D). These differences could be explained by the presence of CD44 on the surface of C28/I2 cells, which is the main receptor for HA and therefore induces a higher binding capacity [27,28]. This was confirmed by blocking CD44 receptors using antibodies and hyaluronic acid, as described below.

#### 3.3.2. NPs’ Uptake by Chondrocytes 

**Figure 6:**
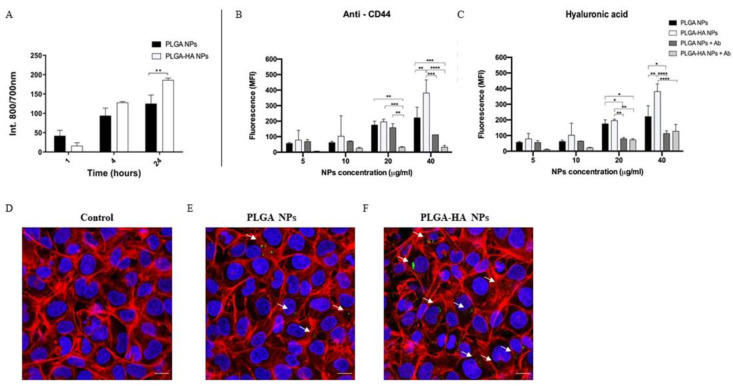
Measurement and visualization of C28/I2 cellular uptake of NPs. (**A**) In vitro cellular uptake of PLGA NPs and PLGA-HA NPs. The C28/I2 cells were incubated at 1, 4, and 24 h with a NP concentration of 40 µg/mL (*n* = 3). (**B**,**C**) In vitro cellular uptake of PLGA NPs and PLGA-HA NPs characterized by FACS. The C28/I2 cells were incubated for 24 h with NP concentrations of 5, 10, 20, and 40 μg/mL. (**B**) The cells were previously treated with anti-CD44 antibody and (**C**) with hyaluronic acid. (**D**–**F**) Confocal microscopy images. (**D**) Control, consisting of cells without NPs, € PLGA NPs and (**F**) PLGA-HA NPs. Phalloidin-TRITC (red), cell nucleus with DAPI (blue), and FITC NPs (green arrows). The images were made with Leica SP8 confocal microscopy at 60× magnification. White bar indicates scale bar at 10 µm. Indicates statistically significant difference (* to *p* < 0.01; ** to *p* < 0.05; *** to *p* < 0.001; **** to *p* < 0.0001).

To assess the evolution of the NP–cell interaction, its fluorescence intensity was measured at three time points after incubating C28/I2 cells with NPs (Figure 6A). Cell cultures incubated with PLGA and PLGA-HA NPs showed an increasing absorption with time, and the highest value was observed at 24 h of incubation. No significant differences were observed between PLGA NPs and PLGA-HA NPs after 1 and 4 h of incubation. However, after 24 h of incubation, cells incubated with PLGA-HA NPs expressed a significantly higher fluorescence intensity than PLGA NPs. This is probably because the modified PLGA-HA NPs are more extensively internalized into chondrocytes through CD44 receptor-mediated endocytosis [29].

To confirm the above results and further investigate site specificity, two additional uptake assays were performed and characterized by flow cytometry. In the first experiment, CD44 receptors were blocked using an antibody against CD44 (Figure 6B), and in the second experiment, CD44 receptors were blocked with HA (Figure 6C). Indeed, the data confirmed the role of CD44 in particle uptake (Figure 6B,C). In cells exposed to PLGA-HA NPs (with either the antibody or HA pretreatment), reduced PLGA-HA NP uptake was inhibited, although at lower concentrations this effect was not significant, probably due to the low average absorption (Figure 6B,C, respectively). PLGA-NPs uptake was not affected by anti-CD44 treatment (Figure 6B). However, pretreatment of cells with HA prevented uptake of both types of particles.

To visualize the internalization of the NPs and their localization in the chondrocytes, confocal microscopy was used to visualize the cells after 24 h of incubation. Combined images and z-stack videos (Supplementary Materials, Video S2) suggested that PLGA and PLGA-HA NPs crossed the cell membrane (Figure 6D–F), while HA-modified NPs (Figure 6F) are more concentrated, compared to PLGA NPs. Taken together, these results are a proof-of-concept that NPs designed for target drug delivery induce higher uptake compared to undesigned NPs.

#### 3.3.3. NPs’ Retention in Mice Joint (In Vivo Study)

**Figure 7:**
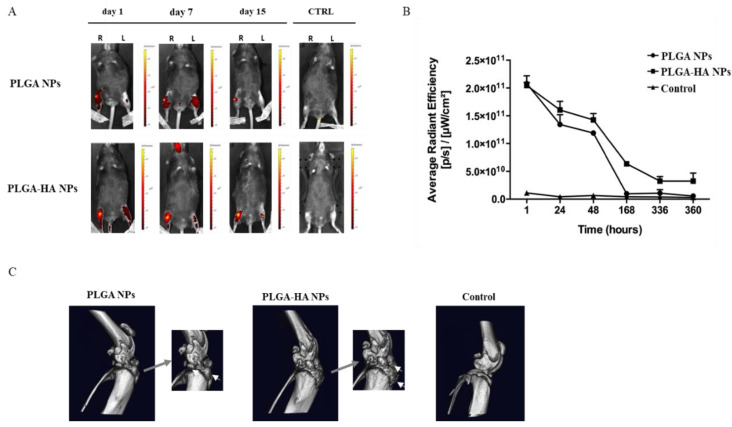
(**A**) In vivo retention of PLGA NPs and PLGA-HA NPs loaded with NIR dye for 15 days after IA injection in the right knee. The pictures depict a representative mouse for each NP treatment group at 3 different time points. From left to right, after 1, 7, and 15 days of NPs intra-articular injection. (**B**) The in vivo retention was measured as the average radiation intensity of each NP treatment over time, at 6 time points, in the OA knee. The control curve represents free dye injected intra-articularly and was used as a baseline comparison with the NPs. In vivo retention of PLGA NPs and PLGA-HA NPs loaded with gold and co-loaded with NIR dye followed for 15 days after IA injection in the right knee. (**C**) µCT scan of knee joints showing a 3D view of the bone surface. From left to right, representative pictures of knee treated with PLGA NPs, PLGA-HA NPs, and healthy knee (negative control) (*n* = 3).

To study NP retention in knee joints, fluorescent PLGA and PLGA-HA NPs were injected intra-articularly into the right knee of mice. In all injected knees, the fluorescent signal obtained with IVIS was clearly visible 15 days after IA injection (Figure 7A), which was quantified in Figure 7B. This result suggested that PLGA NPs and modified PLGA-HA NPs are suitable nanosystems for drug delivery in the knee joint.

After sacrificing the mice, their limbs were collected and analyzed by μCT scans (Figure 7C). Fluorescent PLGA NPs and gold-co-encapsulated PLGA-HA NPs were used as a contrast agent to visualize the presence of NPs in the knee joint of mice. Both types of NPs accumulated in the joint tissue of treated mice, while, as expected, the control group did not show any visual signs of NPs. PLGA-HA NPs showed higher accumulation compared to PLGA NPs. Gold detection was recorded by μCT scans on film (Supplementary Materials, Video S3).

#### 3.3.4. The Target Specificity of NPs in Human Cartilage Explants 

**Figure 8:**
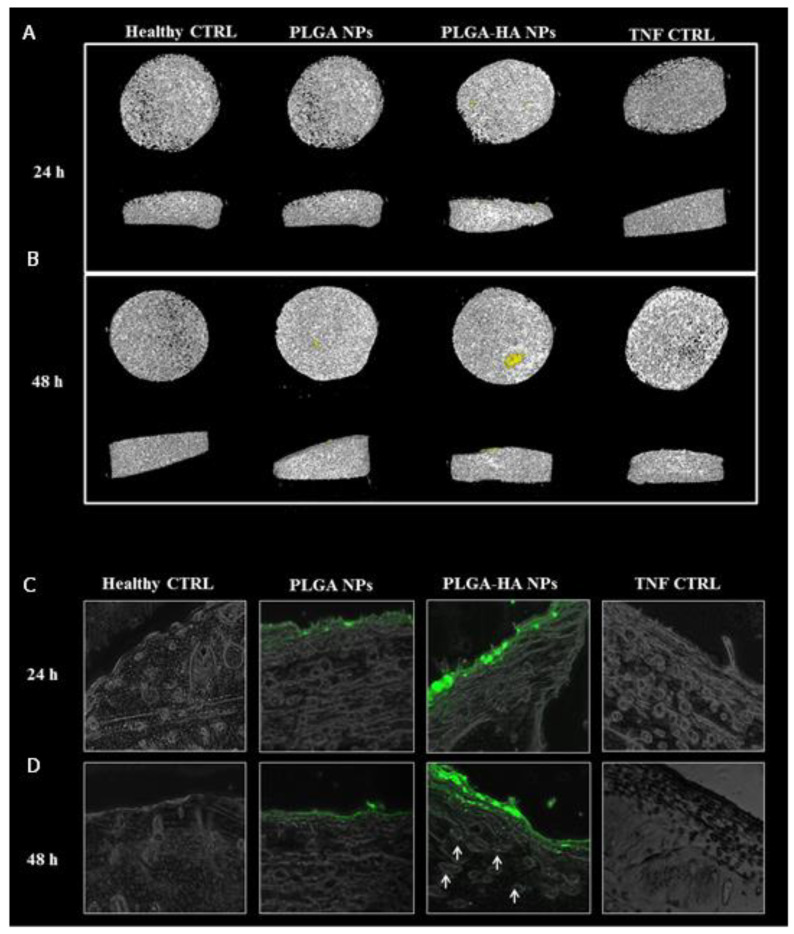
Human cartilage explant incubated with NPs. (**A**) µCT scan of human cartilage and NPs after 24 h of incubation. (**B**) µCT scan of human cartilage and NPs after 48 h of incubation. (**C**) Fluorescent images of human cartilage and NPs after 24 h of incubation at 37 °C. (**D**) Fluorescent images of human cartilage and NPs after 48 h of incubation. (**A**–**D**) From left to right, healthy cartilage (with no TNF treatment), cartilage incubated with TNF and PLGA NPs, cartilage incubated with TNF and PLGA-HA NPs, and cartilage treated with TNF only.

Fluorescent NIR-loaded and gold-coloaded PLGA and PLGA-HA NPs were incubated for 24 and 48 h with TNF-stimulated human cartilage explants (Figure 8). After 24 and 48 h, the cartilage explants were washed in PBS and scanned using computed tomography (Figure 8A,B). The PLGA-HA NPs µCT image clearly showed NPs’ accumulation compared to the PLGA NPs image (in yellow). To confirm the results obtained by µCT scanning, the imaged cartilage explants were sectioned and measured by fluorescence microscopy (Figure 8C,D). The images showed fluorescent NPs on the cartilage surface. Higher fluorescence intensity was observed for PLGA-HA NPs compared to PLGA NPs, probably due to the ability of modified PLGA-HA NPs to bind to the cartilage surface. Furthermore, at 48 h, deep penetration to the thickness of human cartilage explants by PLGA-HA NPs was found (Figure 8D), whereas PLGA NPs did not penetrate cartilage. These results confirm that PLGA-HA NPs can successfully bind to and penetrate human cartilage explants and therefore constitute an attractive avenue for future studies in the search for more efficient treatments for OA.

## 4. Conclusions

In this study, a PLGA-HA copolymer was synthesized and formulated as nanoparticles. Neither PLGA NPs nor PLGA-HA NPs were cytotoxic to chondrocytes, and they were efficiently internalized. In vitro and ex vivo studies showed the higher ability of PLGA-HA NPs to bind to cartilage compared to non-targeted PLGA NPs. Fluorescent NPs encapsulated with NIR dye and co-encapsulated with gold as a contrast agent on µ-computed tomography were successfully visualized by optical imaging in vivo in mouse knee and ex vivo in human explants. In this way, drug-loaded PLGA NPs and PLGA-HA NPs could be monitored in vivo during drug treatment using non-invasive techniques. In conclusion, future research on OA nanotherapy should include PLGA-HA NPs as a targeted drug-delivery system.

## Figures and Tables

**Table 1:** NPs size, PDI, ζ-potential, and NIR dye, gold, and FITC dye drug encapsulation efficiency (EE) of PLGA NPs and PLGA-HA NPs.

NPs	Particle Size (nm)	PDI	ζ-Potential (mV)	EE% (NIR)	EE% (Gold)	EE% (FITC)
PLGA	228 ± 1	0.31 ± 0.05	−15 ± 2	84.3	16.1	24.6
PLGA-HA	200 ± 2	0.6 ± 0.2	−23 ± 2	65.3	8.4	10.6

## Data Availability

Not applicable.

## Supplementary Materials

The following supporting information can be downloaded at: https://www.mdpi.com/article/10.3390/nano12132248/s1, Video S1: µ-CT scan video of PLGA NPs and PLGA-HA NPs loaded with NIR dye and gold; Video S2: Confocal microscopy z-stacks videos. A) Control consisting in cells without NPs, B) PLGA NPs and C) PLGA-HA NPs. Phalloidin-TRITC (red), cell nucleus with DAPI (blue) and FITC NPs (green). The z-stacks were made with Leica SP8 confocal microscopy at 60X magnification; Video S3: µ-CT scan video A) Right knee control mouse and B) representative video of right knee mouse treated with PLGA-HA NPs after 15 days of NPs injection.
