# Supplementary material for: PLGA Nanoparticles Grafted with Hyaluronic Acid to Improve Site-Specificity and Drug Dose Delivery in Osteoarthritis Nanotherapy
Source: Nanomaterials (Basel). 2022 Jun 30;12(13):2248. doi: 10.3390/nano12132248 (PMC9268068; doi:10.3390/nano12132248)
Supplement: Supplementary file 1 [file nanomaterials-12-02248-s001.zip › nanomaterials-1432352-supplementary.pptx]

## Slide 1
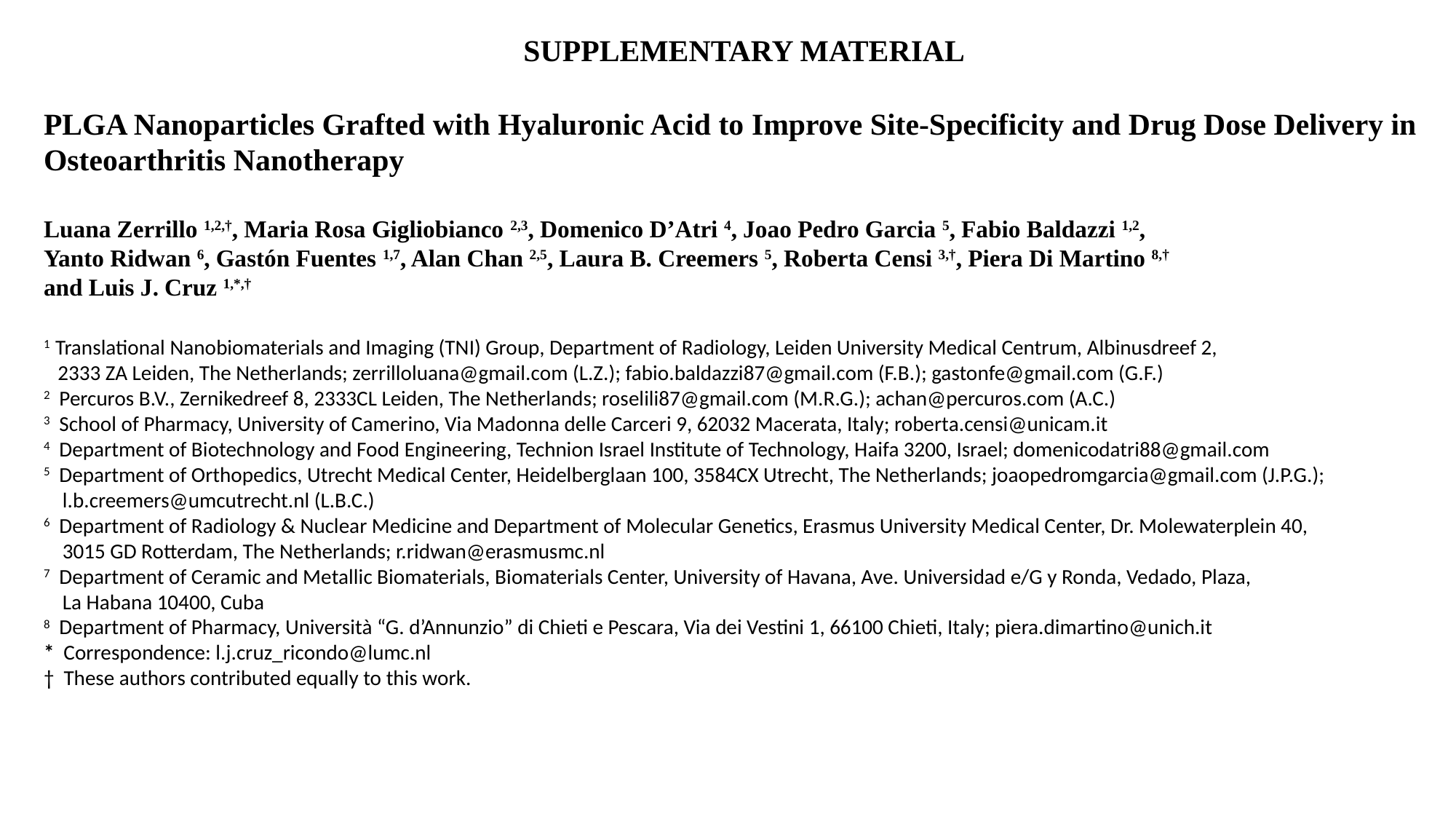

SUPPLEMENTARY MATERIAL
PLGA Nanoparticles Grafted with Hyaluronic Acid to Improve Site-Specificity and Drug Dose Delivery in Osteoarthritis Nanotherapy
Luana Zerrillo 1,2,†, Maria Rosa Gigliobianco 2,3, Domenico D’Atri 4, Joao Pedro Garcia 5, Fabio Baldazzi 1,2,
Yanto Ridwan 6, Gastón Fuentes 1,7, Alan Chan 2,5, Laura B. Creemers 5, Roberta Censi 3,†, Piera Di Martino 8,†
and Luis J. Cruz 1,*,†
1 Translational Nanobiomaterials and Imaging (TNI) Group, Department of Radiology, Leiden University Medical Centrum, Albinusdreef 2,
 2333 ZA Leiden, The Netherlands; zerrilloluana@gmail.com (L.Z.); fabio.baldazzi87@gmail.com (F.B.); gastonfe@gmail.com (G.F.)
2 Percuros B.V., Zernikedreef 8, 2333CL Leiden, The Netherlands; roselili87@gmail.com (M.R.G.); achan@percuros.com (A.C.)
3 School of Pharmacy, University of Camerino, Via Madonna delle Carceri 9, 62032 Macerata, Italy; roberta.censi@unicam.it
4 Department of Biotechnology and Food Engineering, Technion Israel Institute of Technology, Haifa 3200, Israel; domenicodatri88@gmail.com
5 Department of Orthopedics, Utrecht Medical Center, Heidelberglaan 100, 3584CX Utrecht, The Netherlands; joaopedromgarcia@gmail.com (J.P.G.);
 l.b.creemers@umcutrecht.nl (L.B.C.)
6 Department of Radiology & Nuclear Medicine and Department of Molecular Genetics, Erasmus University Medical Center, Dr. Molewaterplein 40,
 3015 GD Rotterdam, The Netherlands; r.ridwan@erasmusmc.nl
7 Department of Ceramic and Metallic Biomaterials, Biomaterials Center, University of Havana, Ave. Universidad e/G y Ronda, Vedado, Plaza,
 La Habana 10400, Cuba
8 Department of Pharmacy, Università “G. d’Annunzio” di Chieti e Pescara, Via dei Vestini 1, 66100 Chieti, Italy; piera.dimartino@unich.it
* Correspondence: l.j.cruz_ricondo@lumc.nl
† These authors contributed equally to this work.

## Slide 2
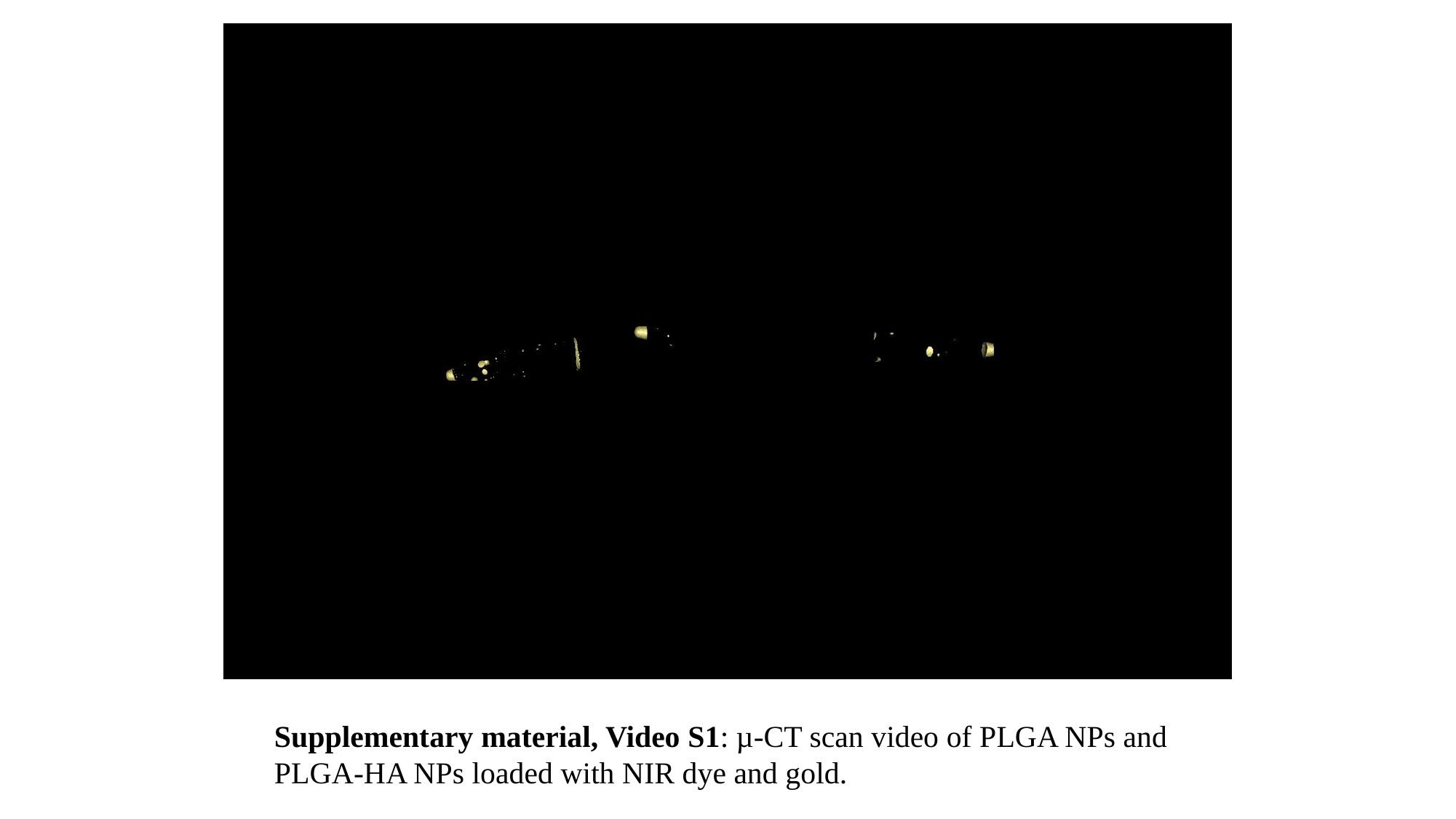

Supplementary material, Video S1: µ-CT scan video of PLGA NPs and PLGA-HA NPs loaded with NIR dye and gold.

## Slide 3
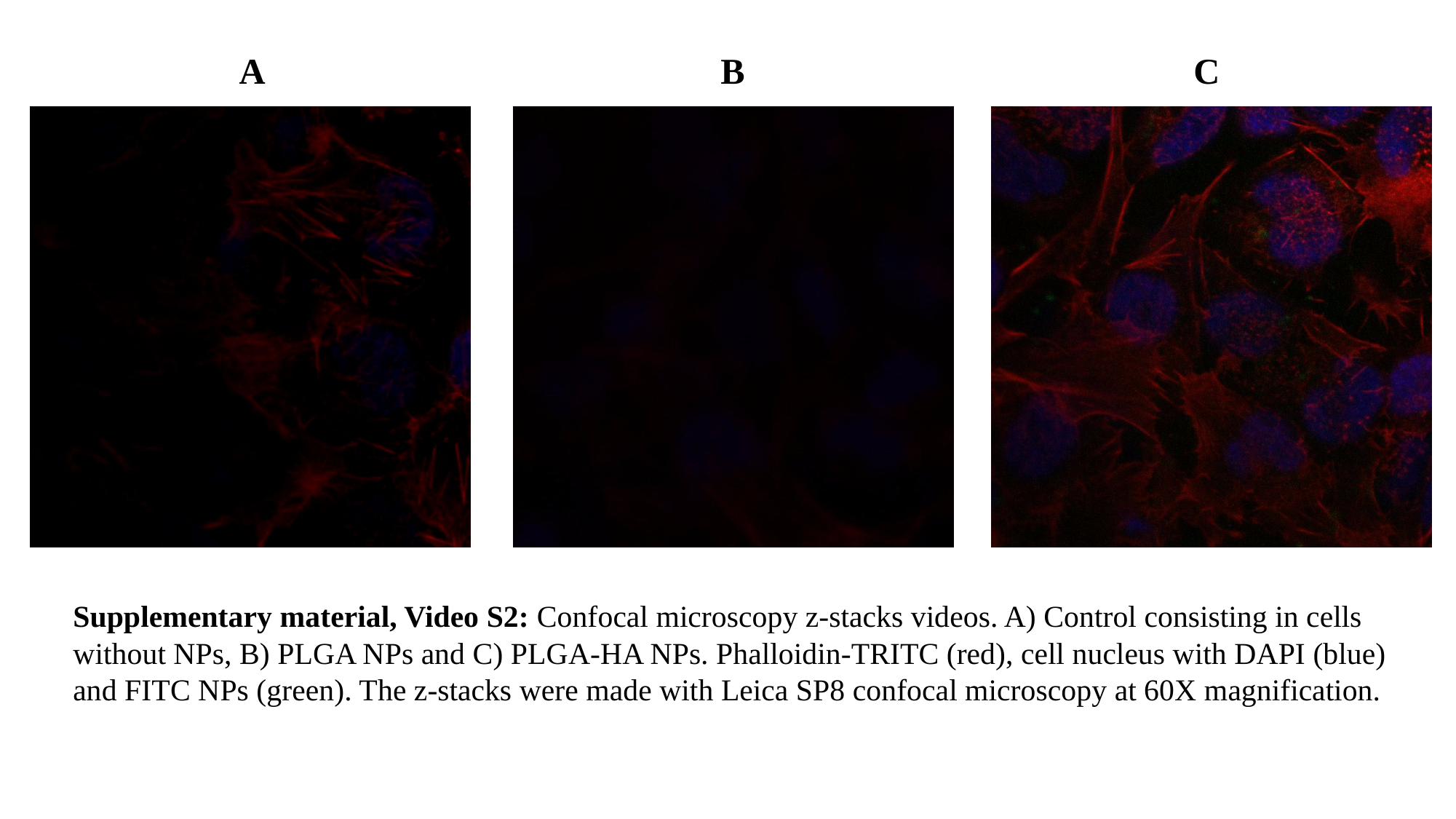

C
B
A
Supplementary material, Video S2: Confocal microscopy z-stacks videos. A) Control consisting in cells without NPs, B) PLGA NPs and C) PLGA-HA NPs. Phalloidin-TRITC (red), cell nucleus with DAPI (blue) and FITC NPs (green). The z-stacks were made with Leica SP8 confocal microscopy at 60X magnification.

## Slide 4
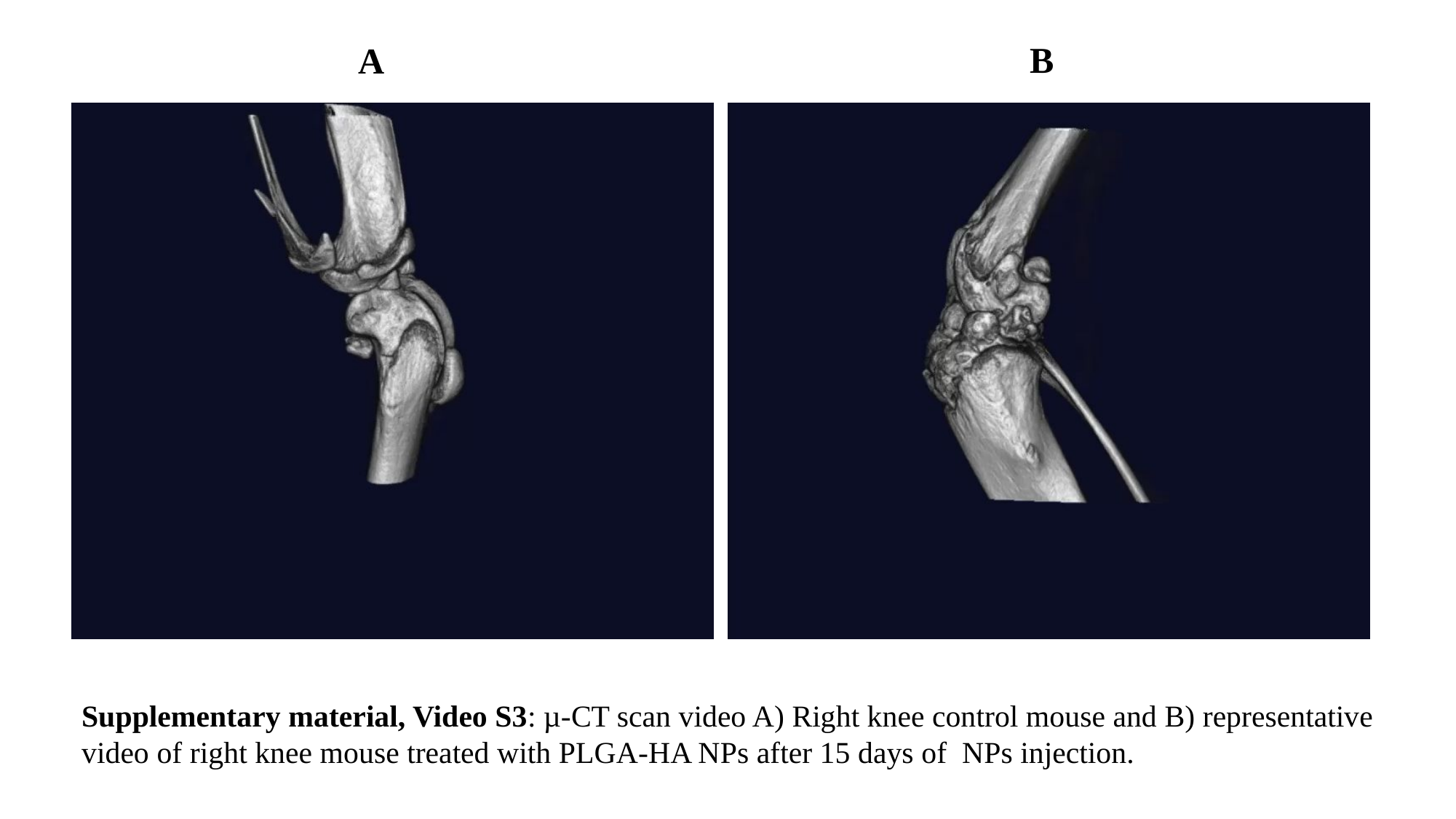

B
A
Supplementary material, Video S3: µ-CT scan video A) Right knee control mouse and B) representative video of right knee mouse treated with PLGA-HA NPs after 15 days of NPs injection.
